# Medical Society of the County of Erie

**Published:** 1904-08

**Authors:** Franklin C. Gram


### SOCIETY PROCEEDINGS.

### Medical Society of the County of Erie.

*Semiannual Meeting.*

Reported by FRANKLIN C. GRAM, M. D., Secretary.

The eighty-third semiannual meeting of the Medical Society of the County of Erie was held in the rooms of the Society of Natural Sciences, Buffalo, Tuesday, June 28, 1904, under the presidency of Dr. William C. Krauss, who took the chair at 11 o'clock a. m.

The minutes of the annual meeting and of the special meeting, held April 9, 1904, to ratify the action of the joint committee consolidating the medical society and the medical association of the state, were read and approved.

Dr. William Warren Potter, chairman of the committee on membership, presented the names of Dr. Erwin W. Buffum, of East Aurora, and Dr. Myrtle Lothrop Massey, of Buffalo, as applicants for membership. He recommended the election of Dr. Buffum, and that the application of Dr. Massey lie on the table until proper credentials were submitted. His recommendations were adopted by the society.

Dr. John H. Grant read a paper on

THE ADULTERATION OF FOOD SUPPLIES, which was discussed by Drs. Edward Clark, A. L. Benedict, and John D. McPherson.

Dr. Frank Van Fleet, of New York, was to have presented the next paper, but sent his regrets through the president.

Dr. Sidney A. Dunham then gave an address on

X-RAY THERAPEUTICS, presenting several interesting patients and photographs. Drs. A. W. Bayliss and S. S. Green discussed the subject and some interesting points were developed.

The society adjourned and in the afternoon a boat ride down the river, with dinner at the Island Club House, afforded a pleasant afternoon and evening for nearly one hundred members.

